# Selection of the Optimal Algorithm for Real-Time Estimation of Beta Band Power during DBS Surgeries in Patients with Parkinson's Disease

**DOI:** 10.1155/2017/1512504

**Published:** 2017-12-24

**Authors:** Ángeles Tepper, Mauricio Carlos Henrich, Luciano Schiaffino, Alfredo Rosado Muñoz, Antonio Gutiérrez, Juan Guerrero Martínez

**Affiliations:** ^1^Laboratory of Rehabilitation Engineering, National University of Entre Ríos, Oro Verde, Argentina; ^2^Group for Digital Design and Processing (GDDP), ETSE, Department of Electronic Engineering, University of Valencia, Valencia, Spain; ^3^Functional Neurosurgery Unit, La Fe Hospital, Valencia, Spain

## Abstract

Deep Brain Stimulation (DBS) is a surgical procedure for the treatment of motor disorders in patients with Parkinson's Disease (PD). DBS involves the application of controlled electrical stimuli to a given brain structure. The implantation of the electrodes for DBS is performed by a minimally invasive stereotactic surgery where neuroimaging and microelectrode recordings (MER) are used to locate the target brain structure. The Subthalamic Nucleus (STN) is often chosen for the implantation of stimulation electrodes in DBS therapy. During the surgery, an intraoperative validation is performed to locate the dorsolateral region of STN. Patients with PD reveal a high power in the *β* band (frequencies between 13 Hz and 35 Hz) in MER signal, mainly in the dorsolateral region of STN. In this work, different power spectrum density methods were analyzed with the aim of selecting one that minimizes the calculation time to be used in real time during DBS surgery. In particular, the results of three nonparametric and one parametric methods were compared, each with different sets of parameters. It was concluded that the optimum method to perform the real-time spectral estimation of beta band from MER signal is Welch with Hamming windows of 1.5 seconds and 50% overlap.

## 1. Introduction

Parkinson's Disease (PD) is a common neurodegenerative disorder. Many successful pharmacological therapies and strategies have been developed to treat both the motor and nonmotor manifestations of PD. However, as PD progresses, it often becomes difficult to treat, typically because of motor complications. In these cases, Deep Brain Stimulation (DBS) is a therapy used to treat PD. Most recently, DBS is also being used in early stages of PD [1].

**Figure 1:**
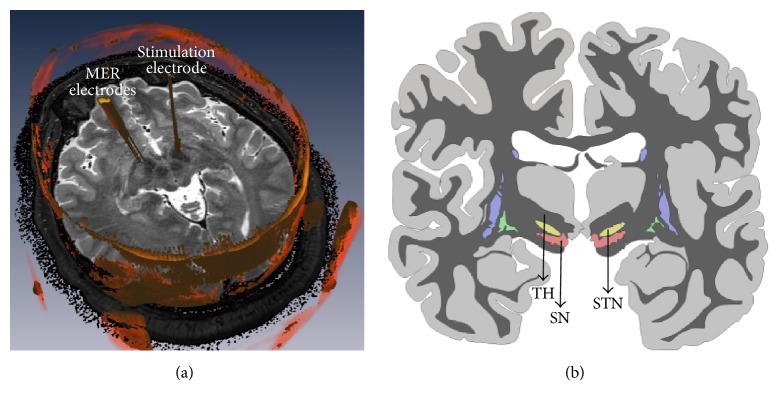
(a) MER and stimulation electrodes in STN during a DBS surgery. (b) Basal nuclei of the brain: TH: Thalamus, STN: Subthalamic Nucleus, and SN: Substantia Nigra.

DBS involves the application of controlled electrical stimuli to a given brain structure by implanted stimulation electrodes. The implantation of the electrodes for DBS is performed by a minimally invasive stereotactic surgery where neuroimaging and microelectrode recordings (MER) are used to locate the target brain structure (Figure 1).

Neuroimaging studies, both Computerized Tomography (CT) and Magnetic Resonance Images (MRI), are used for surgery planning and validation of the implantation site. MER are obtained by recording the neuronal electrical activity using micro EEG technique during implantation surgery and they are used to perform an intraoperative validation of the electrode position. MER signals recorded during surgery are the sum of a variety of signals generated by several neural processes and elements. The extracellular activity captured by MER can be divided into three categories: Local Field Potential (LFP) < 300 Hz, Multi-Unit Activity (MUA) > 300 Hz, and single-unit activity.

For PD, the Subthalamic Nucleus (STN) is often chosen as the target brain structure (Figure 1) [2]. During implantation surgery, an intraoperative validation is performed to locate the dorsolateral region of STN (Figure 1). For these validations, temporal characteristics of MER are used. That is, in order to locate the STN, MER signals are visually analyzed and it is sought to recognize the location where the electrode is located at each moment of the implantation surgery. This validation method requires a highly trained medical team.

Several researchers ([3–5]) demonstrated that STN's MER in humans with PD reveal a high power in the *β* band (frequencies between 13 Hz and 35 Hz), mainly in the dorsolateral region of that subcortical nucleus. In addition, this is the same region providing optimal therapeutic benefits for patients with PD who undergo DBS in the STN [4]. Therefore, research has been carried out to obtain frequency information, which could be valuable to locate the electrode implantation site [6]. Performing signal processing in the operating room to retrieve frequency information would imply having a complementary tool to those currently used, providing to the medical team more information for the selection of the best stimulation site.

From the mathematical point of view, in order to be able to retrieve frequency information from MER, and since the signals are random and only one segment of them is available, an estimation of the power spectrum must be considered [7]. There are different methods for power spectrum density (PSD) estimation of a random signal. In the literature, Welch's algorithm with 1 s Hamming window and 50% overlap is mostly used as the method for PSD estimation of MER signals ([5, 8–12]).

However, for *β* band power detection in real time during DBS surgery, it is necessary to explore algorithms that minimize PSD calculation time. In this work, different PSD methods were analyzed with the purpose of selecting the one that allows the calculation of the *β* band power of MER signals in real time during DBS electrodes implantation surgery.  Section 2 describes the used data obtained from patients who were implanted with a brain stimulator and it details the followed signal processing methodology.  Section 3 describes the obtained results. Finally, discussion and conclusions are given in Sections 4 and 5, respectively.

## 2. Materials and Methods

### 2.1. Patients and Data Collection

For this study, MER recordings obtained from bilateral surgery performed on 9 patients with PD undergoing the implantation of stimulation electrodes for DBS in the STN were used. The surgical interventions took place at La Fe Hospital, in Valencia, Spain.

The recordings were obtained with the “MicroGuide” system (AlphaOmega Engineering, Nazareth, Israel). Neurophysiological activity was recorded through polyamide-coated tungsten microelectrodes (Alpha Omega). The signal was amplified 10000 times and it was filtered with a bandpass filter between 200 and 6000 Hz, using a 4th order Butterworth filter for the low cutoff frequency and 2nd order for the high cutoff frequency. The sampling frequency was 12 kHz, and a 12-bit analog/digital converter was used.

Filtering the recordings with a high-pass filter with cutoff frequency at 200 Hz implies that these signals are MUA and not LFP. This selection of filters, however, is helpful to avoid the recording of electrical noise in the operating room [5]. Despite the fact that the recorded signals do not include low frequencies where *β* band is located, Section 3.2 describes the method used to analyze the *β* band.

During electrode implantation surgery, two parallel MERs in each brain hemisphere were moved in small discrete steps of 0.2 mm starting at 8 mm above the calculated target (center of the dorsolateral STN). The recording times in each depth were variable (between 0.43 s and 278.92 s). In the study conducted by [6] it was determined that, when using power spectral density for STN detection, recordings should not be shorter than one second. For this reason, recordings shorter than 1 s were discarded. Thus, recording times between 1.56 s and 278.92 s with a mean of 48.54 s and a standard deviation of 26.94 s were obtained.

### 2.2. Signal Processing

Signal processing and data analysis were performed with MATLAB software V8.5 R2015a (Mathworks, Natick, MA, USA) using* Signal Processing Toolbox*. To compare the results of the different spectral estimators studied, a statistical analysis was performed using MATLAB* Statistics Toolbox*.

#### 2.2.1. Signal Stability

As reported by other authors ([5, 6, 8]), due to surgical conditions such as electrode tip movements and/or neuronal lesions, it is necessary to select the most stable segment from the MER recording at each depth.

To assess signal stability, the strategy outlined by [5] was followed, but adding an energy threshold condition prior to RMS calculation.

First, signals values whose modulus exceeded 150 *μ*V were replaced by zeros (the action potentials have typical values of 60 *μ*V, possibly reaching 100 *μ*V [9], but there were long portions of signals with high energy in some particularly unstable recordings). Then, each signal was divided into consecutive segments of 50 ms and the RMS of each segment was calculated. A portion of the signal was considered stable when the RMS values of all of the segments fulfilled the following condition:

(1) $$\begin{array}{c} {RMS}_{50\text{ ms}}={RMS}_{median}\pm 3SD, \end{array}$$

where SD is the standard deviation of the RMS of all of the 50 ms segments of the signal.

This stability analysis rejects infrequent events, such as glitches (spurious electronic signals caused by peaks in electrical energy) or cell damage, but does not reject oscillatory activity greater than 1 Hz [5]. The longest stable portion of each signal was selected to continue the analysis, discarding the rest of the recording.

#### 2.2.2. Rectification and Filtering

MER signals are produced by the superposition of multiple electrical sources corresponding to several neural processes. Since the acquisition was performed with a high-pass online filter with cutoff frequency at 200 Hz, the signals used in this work are MUA signals; that is, they are composed by background activity and action potentials from neurons close to the recording electrode.

Because of this acquisition strategy, it is impossible to perform a direct frequency analysis in ranges lower than 200 Hz, which are the ones of interest in this study (*β* band frequencies: 13 Hz to 35 Hz).

In [13], it is shown that MER signals acquired using a high-pass filter can recover its low frequency oscillatory components via the extraction of their envelope, since there is a low frequency modulation in the amplitude of the high frequency signals (MUA). In order to perform frequency analysis in lower frequency ranges, this modulation information needs to be retrieved.

In this study, the strategy proposed by [13] was followed. The low frequency envelope can be calculated following a two-step procedure: extraction of the instantaneous power of the signal by the absolute value operator and smoothing using a low-pass filter. In addition, the mean of the envelope signal was subtracted, given that some methods of power spectrum estimation assume that the signal has zero mean. The power spectrum of a nonzero mean signal has a zero frequency pulse; if the mean is relatively large, it may obscure the components of the low amplitude and low frequency spectrum. Despite the fact the estimation is not an exact value, removing the mean value provides better estimation, especially for low frequencies [7].

Summarizing, to obtain the envelope, these steps were followed:

- Full wave rectification: absolute value of each signal sample.
- Extraction of the mean.
- Smoothing to obtain the envelope: 4th order Butterworth low-pass filter: cutoff frequency at 100 Hz.

### 2.3. Power Spectrum Estimation

In studies where the *β* power of MER signals is obtained ([5, 8–12]), there is a systematic use of Welch's method with 1 s Hamming windows and 50% overlap. However, in order to be able to do an intraoperative validation of the electrode position during implantation surgeries, the *β* band power detection must be achieved in real time, that is, the shorter time possible. Thus, it is necessary to explore algorithms that minimize PSD calculation time.

In this work, different PSD methods were analyzed with the purpose of selecting the one that allows the calculation of the *β* band power of MER signals in real time during DBS electrodes implantation surgery.

First, it was decided to compare different power spectrum estimation methods in order to identify other methods that could also be used. For this, after performing spectral estimation with different methods, a statistical analysis was performed to find significant differences among them. Then the computation time of some of the methods was compared.

In all spectrum estimations, parameters were adjusted to achieve a frequency resolution of 0.1 Hz.

#### 2.3.1. Nonparametric Estimation Methods

Nonparametric methods for power spectrum estimation are based exclusively on the available data, without making any assumptions about the system that generates them. Due to the analyzed signals characteristics, it seems appropriate to use such an estimation method.

In this study, three nonparametric techniques of PSD estimation were analyzed, which are described below.

*Periodogram*. This is the most basic nonparametric method based on the calculation of the Fourier Transform. By definition, the periodogram is not a consistent estimator [7]. Therefore, although the actual spectrum of the signal is unknown, it can be assumed that this estimation is not convenient. However, as it is the basis of other methods, it was decided to evaluate its results. 

*Welch's Method*. Dividing the recordings into segments before calculating the periodogram reduces the estimation variance. Moreover, the length of those segments has an impact on the frequency resolution of the estimation [14]. Welch's method is based on this procedure: the overlap of the segments makes it possible to increase the number of segments (reducing variance) without reducing its length in order to not lose resolution. If segments are shorter, the estimation has worse resolution but better (less) variance.

In this study, the results of three different window sizes were compared, always with an overlap of 50%:

- 0.5-second Hamming window.
- 1-second Hamming window. This is the most used window length in the consulted bibliography.
- 1.5-second Hamming window.

*Multitaper Method*. This method reduces variance using different windows but all with the same length of the signal [7]. Since length is not reduced, the total bias is less than that obtained using signal segments. The time-bandwidth (NW) parameter balances this estimator resolution and variance. When NW increases, the variance decreases, but each estimate has higher spectral leakage and the resulting spectral estimator has more bias.

To use this method, it is necessary to define the set of windows used and the NW parameter, which is related to the number of windows. In this work, the DPSS set of windows, proposed by Thomson, was always used. In order to select NW value, the values used in the consulted bibliography ([15, 16]) were taken into account, where typical values were found to be between 2 and 6. It was decided to analyze the results of this estimator with typical extreme values of NW. Thus, the multitaper method was analyzed with NW = 2 and NW = 6.

#### 2.3.2. Parametric Estimation Methods

Although MER signals characteristics do not seem to be described by a simple parametric model as they come from a system of great complexity, an autoregressive model (AR) was decided to be applied.

The order of the AR model provides a balance between bias and variance [17]. For a small order, the spectrum may not be well estimated (large bias) but it will have less variance. For a large order, the spectrum will exhibit lower bias but may have a lot of variances.

*AR Model with Burg Coefficients*. An AR model with the coefficients calculated by the Burg method was chosen. Regarding the order of the model, it was decided to compare AR models of two different orders. Two orders were selected within the margins of the values used in other studies ([18, 19]). The power spectrum was then estimated using AR model, 4th order, and AR model, 15th order.

### 2.4. Statistical Comparison of Spectral Estimators

A statistical analysis was performed to compare the results of the different spectral estimators studied. For this, all of the signals coming from the 28 trajectories were used (each trajectory contained several signals, each of a different recording depth). In total, 1010 signals were used.

Since there were few samples and the distribution of the samples was unknown, it was decided to work with Friedman's nonparametric method. This test was applied to two matrices. In both cases, each column represented a method of power spectral estimation and each row represented one signal, which was recorded at a specific depth, with a specific microelectrode, in a specific hemisphere, and in a specific patient.

In one of the input matrices, each element of the matrix contained the average *β* band power. In the other case, instead of filling the matrix with power values, it was completed with the frequency values of the *β* band in which the highest power was obtained.

To obtain the significance value of the comparison between two methods, an ad hoc method for multiple comparisons, based on the Tukey-Kramer criterion, was used. The level of significance was *α* = 0.05; that is, the confidence interval was 95%.

### 2.5. Computational Cost Comparison

Since the final goal of this work is to apply one of these estimation methods to an intraoperative validation of the stimulation electrode implantation optimal location, it is necessary that signal processing is done in real time. Thus, processing speed is an important factor to consider.

The computation time of four of the estimators were compared, using a computer with an Intel® Core™ i7-6700HQ Processor, 16 GB SDRAM DDR3L, and running Windows 10 home 64 bits. The methods chosen for the calculation of the computational cost were those that are shown to be more adequate for the calculation of the spectral estimation.

## 3. Results

### 3.1. Stability Analysis

**Figure 2:**
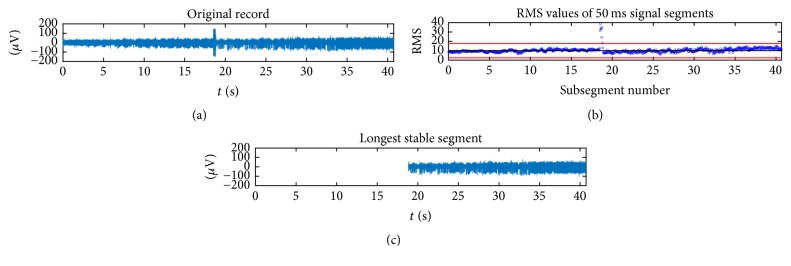
MER recording of patient 2, left hemisphere, central electrode, at a distance from target of 0.524 mm, processed to eliminate spurious data. (a) Original record. (b) RMS values of 50 ms signal segments; the red lines mark the limits corresponding to ±3 standard deviations of the median. (c) Portion of signal considered stable by the algorithm.

An example of the stability analysis is shown in Figure 2. In this case a large increase in the amplitude of the original signal around second 18 can be observed. This segment is considered to be spurious by the algorithm since its RMS value is greater than the upper stability threshold as described above.

As a result of applying this stability analysis to all trajectories, stable portions were obtained from each of them. The length of the stable signals was 21.08 ± 12.18 s, being the minimum and maximum values of 1.55 s and 102.11 s, respectively.

### 3.2. Rectification and Filtering

To obtain the low frequency signal that modulates high frequency in the MER recordings, the method previously explained was applied over the stable segments of the signals.

**Figure 3:**
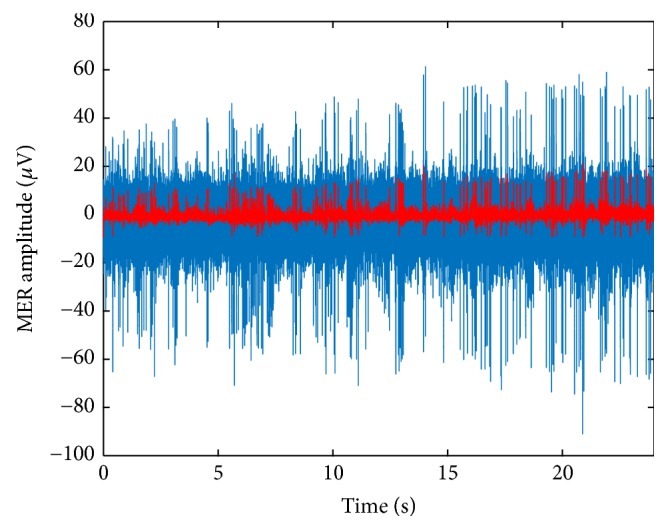
Low frequency modulation signal for patient 8, left hemisphere, central electrode (at a distance from the target of 1636 mm). Blue: stable MER segment. Red: rectified and filtered signal.

As a result of full wave rectification, only positive values of the signal are present. However, the mean of the signal is then subtracted and then some samples may have negative values. Subsequent filtering, with a 4th order Butterworth low-pass filter with cutoff frequency at 100 Hz softens the signal, eliminating some of the original peaks. An example of this processing is illustrated in Figure 3.

### 3.3. Comparison of the Power Spectral Estimation Methods

Since the actual spectrum of the signals is unknown, the results can only be evaluated by doing a comparison between the estimations obtained by each of the methods. That is, the bias of an estimate cannot be assessed without knowledge of the actual spectrum, but the differences in the variability of the different methods or of the same method with different parameters can be seen. Given that in studies where the *β* power of MER signals is obtained ([5, 8–12]), there is a systematic use of Welch's method with 1 s Hamming windows and 50% overlap; that method is taken as a reference in this study.

The goal of this comparison is to identify other methods that could also be used for this application.

#### 3.3.1. Qualitative Comparison

**Figure 4:**
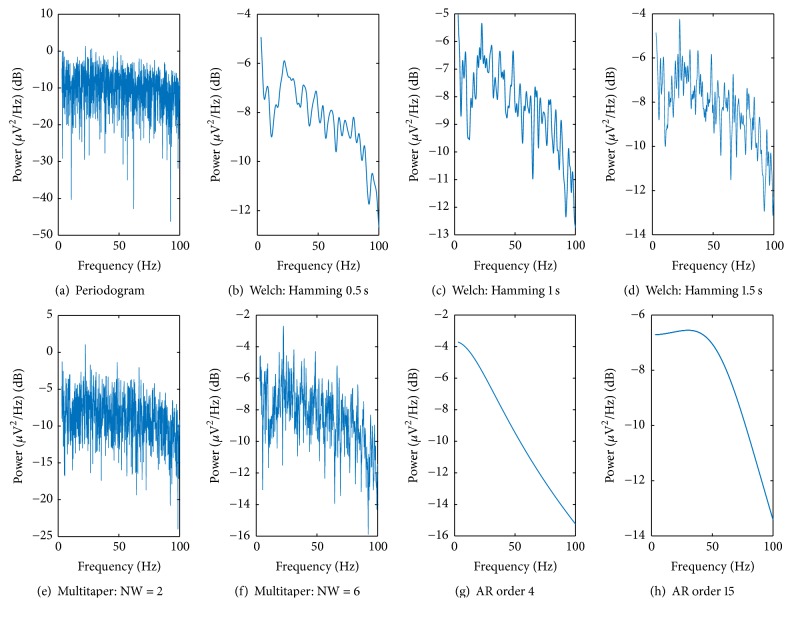
Results of different power spectral estimation methods apply to the signal from patient 5, left hemisphere, central electrode, at a distance from target of 2.235 mm.

In order to perform a qualitative comparison, power spectral estimation of an example signal, calculated with the different methods, is shown in Figure 4. The goal is to make a visual comparison that will be helpful to understand the statistical results that will followed this section. The qualitative comparison provided these results compared with Welch method with Hamming windows of 1 s (Figure 4(d)):

- *Periodogram*.  Figure 4(a) shows the great variability of this estimation method compared with Figure 4(d).
- *Welch's Method*. In the case of 0.5 s Hamming windows (Figure 4(b)), it can be seen that the spectrum is very smooth; that is, it does not present great variability. This agrees with the fact that increasing the number of segments improves the variance of the estimate. However, this improvement implies some loss in frequency resolution. Increasing the length of the segments improves the frequency resolution. With 1.5 s Hamming windows (Figure 4(c)), the spectrum is less smooth, as expected.
- *Multitaper Method*. For multitaper NW = 2, the spectrum (Figure 4(e)) shows some variability. However, when compared with the periodogram, variability in this case is lower. Taking into account the fact that the number of windows used is 2*∗*NW − 1 = 3, it can be thought that these are not sufficient to significantly reduce the variance of the spectral estimate. In case of multitaper NW = 6 (Figure 4(f)), the variance is smaller than NW = 3, which is consistent with the fact that increasing the number of windows improves this feature. The number of windows used is 2*∗*NW − 1 = 11. However, this estimated variance is still considerably higher than that of Welch's estimators.

From the observation of these estimations, and concerning the AR model with Burg coefficients, in the case of the AR model, 4th order, the spectrum (Figure 4(g)) shows how the use of this parametric method implies an excessive simplification of the system under study. The estimated spectrum is completely smooth and has unlimited frequency resolution, but its shape does not match the one given by the other methods, which may indicate that bias is very large (although the actual spectrum is not known, it is more likely that it resembles the ones from nonparametric methods rather than that of this approach). For the AR model, 15th order (Figure 4(h)), the comparison shows an excessive simplification of the system under study.

#### 3.3.2. Quantitative Comparison

Statistical comparisons of the spectral estimations were performed with two different datasets: average power and highest power.

**Figure 5:**
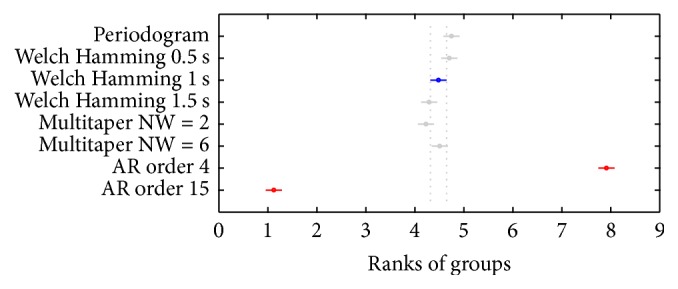
Comparison between Welch's method with 1 s windows and the other estimators. Average power values in the *β* band are compared. Each circle represents the mean of one of the methods and is accompanied by the bars that define a 95% confidence interval. In blue, Welch's method with 1 s windows; in red those methods with which a significant difference has been found; and in gray the methods that are not significantly different to the selected method.

*Comparison of β Band Average Power*.  Figure 5 compares the different PSD estimation methods studied according to their estimations of *β* band average power. In this figure, the value corresponding to Welch's method with 1 s window was selected. It can be seen that this method is significantly different to AR models, but not to the rest of methods.

Table 1 summarizes the *p* values of all of the pairs of the compared methods. It can be seen that several nonparametric PSD methods do not show significant differences among them. The calculation of the periodogram was not significantly different from those of Welch's method with 0.5 s window (*p* = 0.9999), 1 s window (*p* = 0.2284), and multitaper NW = 6 (*p* = 0.3520). On the other hand, for Welch's method, it can be emphasized that when using 1.5 s window, the estimations obtained are not significantly different from those obtained with 1 s window (*p* = 0.6453), but they are different from those obtained with 0.5 s window (*p* = 0.0038). On the other hand, the multitaper estimators did not show significant differences between each other (*p* = 0.1697) and with Welch's method with 1 s window (MTNW = 2: *p* = 0.2741; MTNW = 6: *p* = 1.0000) and 1.5 s window (MTNW = 2: *p* = 0.9992; MTNW = 6: *p* = 0.4884).

With regard to parametric estimators, as expected, after visual inspection of the obtained spectra, the estimations based on AR models presented significant differences with all of the other methods and between each other (*p* = 5.9881*∗*10^−8^).

**Figure 6:**
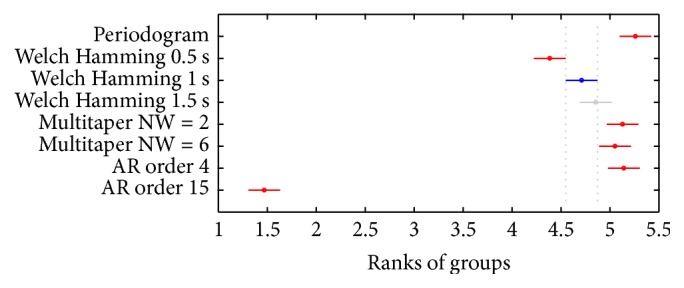
Comparison between 1 s windows Welch's method and the other estimation methods. The values of the frequencies in which the maximum energy in the *β* band is obtained are compared. Each circle represents the mean of one of the methods and is accompanied by the bars that define a 95% confidence interval. In blue, Welch's method with 1 s windows; in red, those methods with which a significant difference has been found; and in gray, the methods that are not significantly different to the selected one.

*Comparison of the β Band Frequency Value with the Highest Power*.  Figure 6 compares the different PSD estimation methods studied according to the frequency of the *β* band in which the highest power appears. In this figure, once again, the value corresponding to Welch's method with 1 s window was selected.

Table 2 summarizes the *p* values of the paired comparisons among all of the methods. In this comparison, the periodogram did not have significant differences with the multitaper methods (MTNW = 2: *p* = 0.9238; MTNW = 6: *p* = 0.5155) and with the 4th order AR model (*p* = 0.9554), but it provided significant differences with all of the others methods. Welch's method with 1 s window was not significantly different from Welch's method with 1.5 s window (*p* = 0.8801), but it was significantly different to all others. The multitaper estimations, as in the previous comparison, did not present significant differences between each other (*p* = 0.9963) nor with Welch's method with 1.5 s window (MTNW = 2: *p* = 0.1680; MTNW = 6: *p* = 0.5895), but it shows differences with Welch's method with 1 s window (MTNW = 2: *p* = 0.0023; MTNW = 6: *p* = 0.0306).

Regarding parametric estimators, as in the previous comparison, AR models presented significant differences between each other (*p* = 5.9881*∗*10^−8^).

#### 3.3.3. Computational Cost Comparison

Since the final goal of this work is to apply one of these methods for intraoperative validation of the implantation's optimal location, it is necessary that signal processing is done in real time. Thus, processing speed is a factor to consider.

The computation time of four of the estimators was compared. The methods chosen for the calculation of the computational cost were those that, from the previous comparisons, showed to be more suitable for the calculation of the spectral estimation, that is, Welch's method with 1 s and 1.5 s window and multitaper method with NW = 2 and NW = 6. The evaluation of the computation time was done in the signals from patient 1, left hemisphere, posteromedial electrode, whose duration was 27.9987 ± 11.9316 s.

The computational times required by each of these methods for the PSD estimation are summarized in Table 3.

## 4. Discussion

Comparisons were done in order to choose the best method to perform an intraoperative validation that takes into account frequency characteristics of MER recordings from PD patients to be performed in real time.

First, it was necessary to compare different power spectrum estimation methods in order to identify methods that could be used for this application. Since the actual power spectrum of the analyzed signals is unknown, it is not possible to evaluate an estimator by how close it is to the ideality. Therefore, a comparison was made between different estimators, taking as a reference the mostly used method in the consulted bibliography ([5, 8–12]), which is Welch's method with 1 s Hamming window and 50% overlap.

The bias of an estimate cannot be assessed without knowledge of the real spectrum, but the differences in the variability of the different methods or of the same method with different parameters can be seen. Here parametric and nonparametric methods were considered.

Regarding parametric methods, the results obtained by the AR models confirm the previous assumption that, without making a much deeper study, these are not optimal for processing MER recordings. Their results are not only significantly different from the results of the other methods, but they are also different from each other.

Regarding nonparametric estimates, although qualitative comparisons showed certain morphological similarities between all methods, the quantitative analysis shows that they may be significantly different from each other.

For the particular case of the periodogram, it presented similarities with other nonparametric methods in the comparisons; however, it must be remembered that this is not a consistent estimator and a high variance was observed in the qualitative analysis. Although some of its results may not be significantly different from those of other estimators, we consider that it is not convenient to continue this work with an inconsistent estimator, since other options are available.

On the other hand, regarding the mostly used method in the consulted bibliography (Welch's method with 1 s Hamming windows and 50% overlap), the results of our comparisons show that there are significant differences, at least in one of the two comparisons, with all of the other tested methods, except for Welch's method with 1.5 s windows. This means that it does have significant differences with the case of 0.5 s windows, but—taking into account the qualitative comparison—a reason for these differences can be found. Welch's method with 1 s window is a method with little variance but sufficient resolution so as not to lose all of the peaks. Estimations obtained with the same method but other window sizes are morphologically similar, but the 0.5 s windows provided a spectrum with no peaks. Having shorter windows allows the method to further soften the spectrum, but this can lead to missed peaks that should be taken into account. This could explain why the results with 0.5 s windows do not match those of the other window sizes when comparing the frequency values in which the maximum power in the *β* band is obtained.

So, for Welch's method, the results indicate that 1 s and 1.5 s windows provide results that are not significantly different from each other. Since this method with 1 s windows is the most used in the consulted bibliography and given that there are no significant differences with the one with 1.5 s windows, the possibility of working with one of these estimators indistinctly could be considered.

As for the multitaper method, in first place, these estimators do not present significant differences between them in any of the two comparisons. On the other hand, in the qualitative comparisons, it can be seen that these methods give more variable results than Welch's method. The quantitative comparison reveals that they do not present significant differences with Welch's method with 1.5 s windows. If this result is analyzed taking into account the explanation in the previous paragraph, the use of multitaper methods for the study of MER recordings could also be considered.

According to the statistical analysis performed, the methods that could be considered for PSD estimation in MER recordings are multitaper with NW = 2 and NW = 6 and Welch's with Hamming windows of 1 s and 1.5 s.

However, in order to be able to do an intraoperative validation of the electrode position during implantation surgeries, the *β* band power detection must be achieved in real time, that is, the shorter time possible. Thus, it is necessary to explore algorithms that minimize PSD calculation time.

In order to select a single method, then, the computational cost of each of them was considered. The results show that multitaper methods are computationally more expensive than those of Welch. In addition, the comparison suggests that Welch's method with 1.5 s windows is the fastest: 35% faster than Welch with 1 s windows and 617% faster than Multitaper_NW=2_.

## 5. Conclusion

In this study, a comparison was made between different PSD estimation methods, taking into account a particular application. MER signal processing and particularly its frequency information can serve as an intraoperative validation tool for best electrode placement during DBS electrode implantation surgery in PD patients. The most used method for spectral estimation in the literature is that of Welch with 1 s Hamming windows and 50% overlap.

In this study we compared different spectral estimators and also the computational costs involved were taken into account. Finally, according to the discussion, we propose Welch's method with 1.5 s Hamming windows and 50% overlap as the most appropriate real-time PSD estimator for MER signals of PD patients.

Even though the selection was based on the idea of performing an intraoperative validation in real time, the methods were not applied online. To further test the utility of the selected method, it would be appropriate to generate a hardware set, in which the registration of the signals could be simulated as if it was on the actual operating room, and the *β* band power detection could be achieved in real time, while the signals are being read. Moreover, it would be necessary to ideate a convenient way to show this frequency information, so that it could be easily read and understood by the medical team.

## Figures and Tables

**Table 1:** Statistical results (*p* values) from the multiple comparisons of the PSD methods regarding average *β* band power values. In bold, significant values for *α* = 0.05.

	PGRM	Welch 0.5 s	Welch 1 s	Welch 1.5 s	Mult. 2	Mult. 6	AR 4	AR 15
PGRM	-	0.9999	0.2284	**7.3461 ** **∗** ** 1**0^−4^	**5.2344 ** **∗** ** 1**0^−5^	0.3520	**5.9881 ** **∗** ** 1**0^−8^	**5.9881 ** **∗** ** 1**0^−8^
Welch 0.5 s	0.9999	-	0.4637	**0.0038**	**3.4350 ** **∗** ** 1**0^−4^	0.6205	**5.9881 ** **∗** ** 1**0^−8^	**5.9881 ** **∗** ** 1**0^−8^
Welch 1 s	0.2284	0.4637	-	0.6453	0.2741	1.0000	**5.9881 ** **∗** ** 1**0^−8^	**5.9881 ** **∗** ** 1**0^−8^
Welch 1.5 s	**7.3461 ** **∗** ** 1**0^−4^	**0.0038**	0.6453	-	0.9992	0.4884	**5.9881 ** **∗** ** 1**0^−8^	**5.9881 ** **∗** ** 1**0^−8^
Mult. 2	**5.2344 ** **∗** ** 1**0^−5^	**3.4350 ** **∗** ** 1**0^−4^	0.2741	0.9992	-	0.1697	**5.9881 ** **∗** ** 1**0^−8^	**5.9881 ** **∗** ** 1**0^−8^
Mult. 6	0.3520	0.6205	1.0000	0.4884	0.1697	-	**5.9881 ** **∗** ** 1**0^−8^	**5.9881 ** **∗** ** 1**0^−8^
AR 4	**5.9881 ** **∗** ** 1**0^−8^	**5.9881 ** **∗** ** 1**0^−8^	**5.9881 ** **∗** ** 1**0^−8^	**5.9881 ** **∗** ** 1**0^−8^	**5.9881 ** **∗** ** 1**0^−8^	**5.9881 ** **∗** ** 1**0^−8^	-	**5.9881 ** **∗** ** 1**0^−8^
AR 15	**5.9881 ** **∗** ** 1**0^−8^	**5.9881 ** **∗** ** 1**0^−8^	**5.9881 ** **∗** ** 10** ^**−8**^	**5.9881 ** **∗** ** 1**0^−8^	**5.9881 ** **∗** ** 1**0^−8^	**5.9881 ** **∗** ** 1**0^−8^	**5.9881 ** **∗** ** 1**0^−8^	-

**Table 2:** Statistical results (*p* values) from multiple comparisons of the PSD methods regarding *β* band frequency values in which the maximum power is reached. In bold, significant values for *α* = 0.05.

	PGRM	Welch 0.5 s	Welch 1 s	Welch 1.5 s	Mult. 2	Mult. 6	AR 4	AR 15
PGRM	-	**5.9881 ** **∗** ** 1**0^−8^	**7.4812 ** **∗** ** 1**0^−6^	**0.0037**	0.9238	0.5155	0.9554	**5.9881 ** **∗** ** 1**0^−8^
Welch 0.5 s	**5.9881 ** **∗** ** 1**0^−8^	-	**0.0487**	**3.0526 ** **∗** ** 1**0^−4^	**5.9961 ** **∗** ** 1**0^−8^	**7.1982 ** **∗** ** 1**0^−8^	**5.9913 ** **∗** ** 1**0^−8^	**5.9881 ** **∗** ** 1**0^−8^
Welch 1 s	**7.4812 ** **∗** ** 1**0^−6^	**0.0487**	-	0.8801	**0.0023**	**0.0306**	**0.0014**	**5.9881 ** **∗** ** 1**0^−8^
Welch 1.5 s	**0.0037**	**3.0526 ** **∗** ** 1**0^−4^	0.8801	-	0.1680	0.5895	0.1262	**5.9881 ** **∗** ** 1**0^−8^
Mult. 2	0.9238	**5.9961 ** **∗** ** 1**0^−8^	**0.0023**	0.1680	-	0.9963	1.0000	**5.9881 ** **∗** ** 1**0^−8^
Mult. 6	0.5155	**7.1982 ** **∗** ** 1**0^−8^	**0.0306**	0.5895	0.9963	-	0.9906	**5.9881 ** **∗** ** 1**0^−8^
AR 4	0.9554	**5.9913 ** **∗** ** 1**0^−8^	**0.0014**	0.1262	1.0000	0.9906	-	**5.9881 ** **∗** ** 1**0^−8^
AR 15	**5.9881 ** **∗** ** 1**0^−8^	**5.9881 ** **∗** ** 1**0^−8^	**5.9881 ** **∗** ** 1**0^−8^	**5.9881 ** **∗** ** 1**0^−8^	**5.9881 ** **∗** ** 1**0^−8^	**5.9881 ** **∗** ** 1**0^−8^	**5.9881 ** **∗** ** 1**0^−8^	-

**Table 3:** Computation times for the trajectory corresponding to patient 1, left hemisphere, posteromedial electrode, whose duration is 27.9987 ± 11.9316 s.

Method	Computation times
Welch 1 s windows	1.1137 ± 0.4831 s
Welch 1.5 s windows	0.7253 ± 0.3168 s
Multitaper NW = 2	2.3227 ± 0.8811 s
Multitaper NW = 6	4.4735 ± 1.8008 s
